# Empirical comparisons of heterogeneity magnitudes of the risk difference, relative risk, and odds ratio

**DOI:** 10.1186/s13643-022-01895-7

**Published:** 2022-02-12

**Authors:** Yuxi Zhao, Elizabeth H. Slate, Chang Xu, Haitao Chu, Lifeng Lin

**Affiliations:** 1 Department of Statistics, Florida State University, 411 OSB, 117 N Woodward Avenue, Tallahassee, FL USA; 2 Key Laboratory of Population Health Across Life Cycle, Ministry of Education of the People’s Republic of China, Hefei, Anhui China; 3 Anhui Provincial Key Laboratory of Population Health and Aristogenics, Anhui Medical University, Hefei, Anhui China; 4 Division of Biostatistics, University of Minnesota, Minneapolis, MN USA

## Introduction

In epidemiology and medical research, the choices of effect measures for binary outcomes have been long debated. Common choices include the risk difference (RD), relative risk (RR), and odds ratio (OR). The RD is often considered more heterogeneous than the ratio measures, RR and OR [1, 2]. Nevertheless, the arguments supporting this claim have been challenged [3]. For example, more rejections of homogeneity in hypothesis testing of RDs are expected than those of ORs. This article empirically compares the heterogeneity magnitudes between the RD, RR, and OR.

## Methods

We applied heterogeneity measures to a large Cochrane database of meta-analyses [4]. The Cochrane Library publishes systematic reviews on a wide range of healthcare-related topics. We searched for all Cochrane reviews available online from issue 1 in 2003 to issue 1 in 2020. The search strategy for an older version of the Cochrane database was used in our earlier work [5–7]. In the Cochrane Library, each issue was published monthly, and it included systematic reviews on new topics with formal meta-analyses as well as protocols without formal analyses. An issue may also publish notices to withdraw outdated or flawed reviews and protocols. In this study, we iteratively included all published reviews that reported statistical data in each issue and excluded all withdrawn reviews. In total, we identified 64,929 meta-analyses.

In addition, a Cochrane review could investigate multiple disease outcomes and/or multiple intervention comparisons. Therefore, the meta-analyses within the review may not be independent due to the correlations between outcomes or intervention comparisons. For removing the impact of such potential correlations on heterogeneity, we also conducted sensitivity analyses, which were restricted to the meta-analyses with the largest number of studies from each Cochrane review. A total of 3125 meta-analyses were included in the sensitivity analyses.

We focused on the heterogeneity measure *I* ^2^ and also considered the CV_B_ statistic as a supplemental measure. We reanalyzed each Cochrane meta-analysis and obtained the heterogeneity measures using each effect measure. The RR and OR were analyzed on the logarithmic scale. The *I* ^2^ is widely used and is interpreted as a percentage of total variation due to heterogeneity rather than sampling error [4]. The CV_B_ is the between-study coefficient of variation used for providing further insight into heterogeneity magnitudes; it is calculated as the ratio of the between-study standard deviation *τ* over the absolute value of the overall effect size [8]. In this article, we estimated the between-study variance *τ* ^2^ using both the DerSimonian–Laird (DL) and restricted maximum likelihood (REML) methods; the former is the most popular while the latter is recommended with better statistical performance.

## Results

**Fig. 1:**
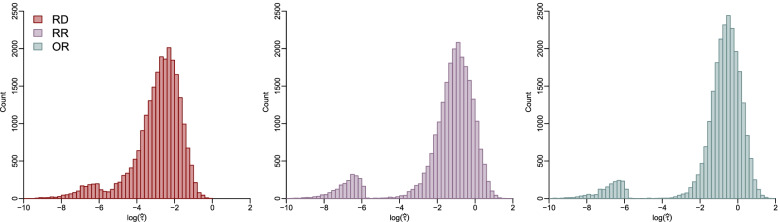
Histograms of between-study standard deviations on a logarithmic scale based on the restricted maximum likelihood method for the RD, RR, and OR. The histograms are restricted to the range from −10 to 2 for $\log \hat{\tau}$

Figure 1 and Fig. S1 present the histograms of $\hat{\tau}$ on a logarithmic scale for the RD, RR, and OR based on the REML and DL estimation methods. Because *τ* that truly equals 0 may not be exactly estimated as 0, depending on the tolerance of the REML algorithm’s convergence, the histograms in Fig. 1 shows small peaks at very small $\hat{\tau}$ values. As the RD, RR, and OR are on different scales, the magnitudes of their corresponding $\hat{\tau}$ may not be directly comparable. In general, the RR and OR led to $\hat{\tau}$ < 0.01 in more meta-analyses than the RD (Table S1).

**Fig. 2:**
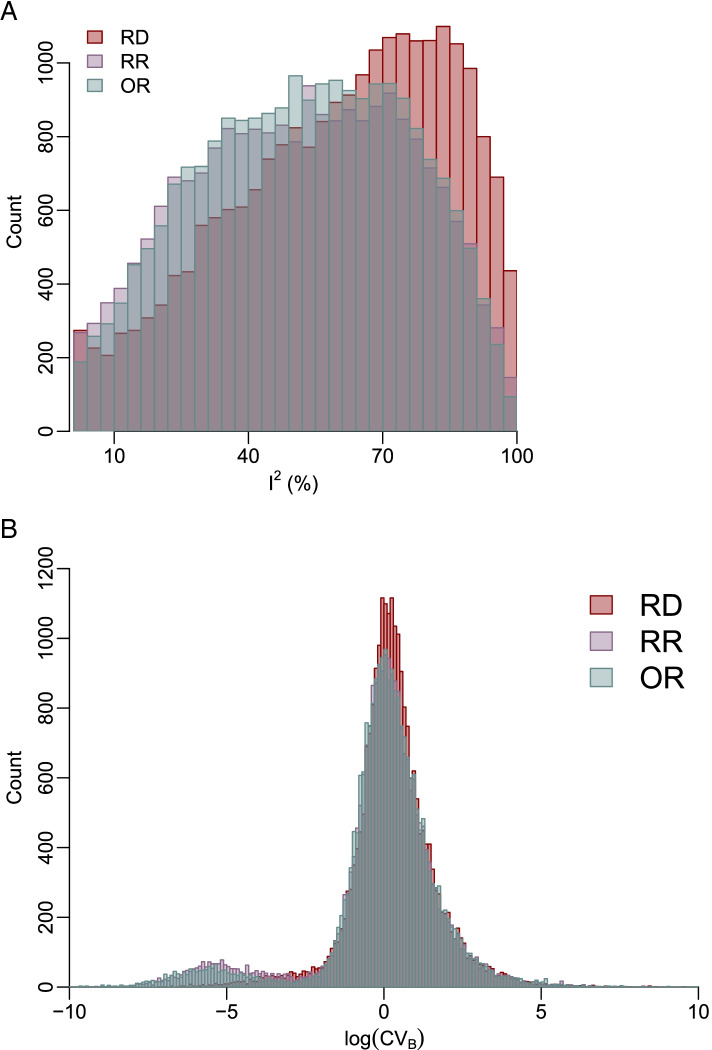
Histograms of *I* ^2^ (**A**) and CV_B_ on a logarithmic scale (**B**) based on the restricted maximum likelihood method for the RD, RR, and OR. **A** Restricted to *I* ^2^ > 1%. **B** Restricted to the range from −10 to 10 for better visualizations

Among the 64,929 Cochrane meta-analyses, 48.09% of RDs had *I* ^2^ = 0% based on the DL method, while about 56% of RRs and ORs had *I* ^2^ = 0%. The REML algorithm failed to converge in a few meta-analyses (≤ 0.22%) and *I* ^2^ was not calculable; for the remainder, 43.56% of RDs had *I* ^2^ = 0%, while about 50% of RRs and ORs had *I* ^2^ = 0%. About 6% of RDs, RRs, and ORs had 0% <*I* ^2^ ≤ 1%; their REML estimates of *τ* were very close, but not exactly equal, to 0. Fewer DL estimates (≤ 0.40%) led to 0% < *I* ^2^ ≤ 1%, while the DL and REML methods produced similar numbers of meta-analyses with 0% ≤ *I* ^2^ ≤ 1% (Table S2). In about 40% of meta-analyses, the RDs’ *I* ^2^ were larger than the RRs’ or ORs’ by over 1%, while in about 10 to 15% of meta-analyses, the RDs’ *I* ^2^ were smaller than the RRs’ and ORs’ by over 1% (Table S3). Based on the *Q* test, there were more meta-analyses (about 10%) with significant heterogeneity for RDs and non-significant heterogeneity for RRs or ORs than meta-analyses (about 1%) with non-significant for RDs and significant heterogeneity for RRs or ORs (Table S4). The RDs’ histogram was right-skewed, with a peak around *I* ^2^ = 70%; the RRs’ and ORs’ histograms were less skewed, with peaks around *I* ^2^ = 50% (Figs. 2A and S2). Table S5 presents the mean and quantiles of *I* ^2^; they were based on 23,966 meta-analyses with *I* ^2^ > 0% for all three measures and both the DL and REML methods to avoid the impact of many *I* ^2^ = 0%. The RDs’ descriptive statistics of *I* ^2^ were noticeably larger than the RRs’ and ORs’.

Categorized by the number of studies, the average study size, and the total number of events in a meta-analysis, RDs continued to have larger *I* ^2^ than RRs and ORs in each category (Fig. S3). The *I* ^2^ slightly decreased as the number of studies increased, consistent with previous findings [9]. It remained nearly unchanged as the average study size increased and noticeably increased as the total number of events increased.

Similar to the trends of *I* ^2^, the histograms in Fig. 2B and S4 indicate that RDs generally had greater CV_B_ values than RRs and ORs. The conclusions regarding CV_B_ by categories of number of studies, average study size, and the total number of events in a meta-analysis were also consistent with those regarding *I* ^2^ (Figure S5).

In sensitivity analyses using the 3125 meta-analyses with the largest number of studies from each review, the histograms’ overall trends were similar to those based on the complete datasets (Figs. S6 and S7).

## Discussion

Our findings consistently supported that the RD seems more heterogeneous than the RR and OR. Yet, large uncertainties in *I* ^2^ may confound these findings. The accuracy of *I* ^2^ may also be questionable in meta-analyses with few studies and/or rare events [10]. In addition, *I* ^2^ has several limitations; for example, it increases as sample sizes increase for the same *τ* ^2^. The CV_B_ overcomes this drawback, while it is also subject to some disadvantages, as it increases rapidly for the overall effect size approaching 0. Nevertheless, they are arguably the appropriate tools with intuitive interpretations available in the current research synthesis literature to compare heterogeneity of measures across different scales. We intend our findings as supporting evidence rather than an assertion about heterogeneity magnitudes.

## Supplementary Information

**Additional file 1: Table S1.** Summary of situations where $\hat{\tau}$ is not calculable or takes very small values (<0.01) among the 64,929 meta-analyses. **Table S2.** Summary of situations where *I*^2^ is not calculable, equals 0%, or takes very small values (≤1%) among the 64,929 meta-analyses. **Table S3.** Comparisons between *I*^2^ of the RD, RR, and OR within the 64,929 meta-analyses. **Table S4.** *Q* test results (with the significance level at 0.05) among the pairs of RD, RR, and OR within the 64,929 meta-analyses. **Table S5.** Summary of descriptive statistics of *I*^2^ (%) among the 23,966 meta-analyses with *I*^2^>0% for all three effect measures based on both the DL and REML methods. **Figure S1.** Histograms of between-study standard deviations on a logarithmic scale based on the DerSimonian–Laird method for the RD, RR, and OR. The histograms are restricted to the range from −8 to 2 for log$\hat{\tau}$. **Figure S2.** Histogram of *I*^2^ based on the DerSimonian–Laird method for the RD, RR, and OR, restricted to *I*^2^>1% for better visualizations. **Figure S3.** Boxplots of *I*^2^ for the RD, RR, and OR categorized by the number of studies (panels a and b), average study size (panels c and d), and total number of events (panels e and f), restricted to *I*^2^>1%. The left panels a, c, and e are based on the DerSimonian–Laird method, and the right panels b, d, and f are based on the restricted maximum likelihood (REML) method. **Figure S4.** Histogram of CV_B_ on a logarithmic scale based on the DerSimonian–Laird method for the RD, RR, and OR. **Figure S5.** Boxplots of CV_B_ on a logarithmic scale for the RD, RR, and OR categorized by the number of studies (panels a and b), average study size (panels c and d), and total number of events (panels e and f). The left panels a, c, and e are based on the DerSimonian–Laird method, and the right panels b, d, and f are based on the restricted maximum likelihood (REML) method. **Figure S6.** Histograms of *I*^2^ for the RD, RR, and OR, restricted to *I*^2^>1% for better visualizations, among the meta-analyses with the largest number of studies from each Cochrane review. Panel a is based on the DerSimonian–Laird method, and panel b is based on the restricted maximum like-lihood (REML) method. **Figure S7.** Histograms of CV_B_ on a logarithmic scale for the RD, RR, and OR among the meta-analyses with the largest number of studies from each Cochrane review. Panel a is based on the DerSimonian–Laird method, and panel b is based on the restricted maximum likelihood (REML) method.

## Data Availability

The datasets and code for this study are available upon reasonable request from the corresponding author.

## References

[1] Engels EA, Schmid CH, Terrin N, Olkin I, Lau J (2000). Heterogeneity and statistical significance in meta-analysis: an empirical study of 125 meta-analyses. Stat Med.

[2] Deeks JJ (2002). Issues in the selection of a summary statistic for meta-analysis of clinical trials with binary outcomes. Stat Med.

[3] Poole C, Shrier I, VanderWeele TJ (2015). Is the risk difference really a more heterogeneous measure?. Epidemiology.

[4] Higgins JPT, Thompson SG, Deeks JJ, Altman DG (2003). Measuring inconsistency in meta-analyses. BMJ.

[5] Lin L, Chu H, Murad MH, Hong C, Qu Z, Cole SR, Chen Y (2018). Empirical comparison of publication bias tests in meta-analysis. J Gen Intern Med.

[6] Ma X, Lin L, Qu Z, Zhu M, Chu H (2018). Performance of between-study heterogeneity measures in the Cochrane Library. Epidemiology.

[7] Lin L, Shi L, Chu H, Murad MH (2020). The magnitude of small-study effects in the *Cochrane Database of Systematic Reviews*: an empirical study of nearly 30 000 meta-analyses. BMJ Evid Based Med.

[8] Takkouche B, Khudyakov P, Costa-Bouzas J, Spiegelman D (2013). Confidence intervals for heterogeneity measures in meta-analysis. Am J Epidemiol.

[9] IntHout J, Ioannidis JPA, Borm GF, Goeman JJ (2015). Small studies are more heterogeneous than large ones: a meta-meta-analysis. J Clin Epidemiol.

[10] Xu C, Furuya-Kanamori L, Zorzela L, Lin L, Vohra S (2021). A proposed framework to guide evidence synthesis practice for meta-analysis with zero-events studies. J Clin Epidemiol.

