# Supplementary material for: Empirical comparisons of heterogeneity magnitudes of the risk difference, relative risk, and odds ratio
Source: Syst Rev. 2022 Feb 12;11:26. doi: 10.1186/s13643-022-01895-7 (PMC8840324; doi:10.1186/s13643-022-01895-7)
Supplement: Supplementary file 1 — Additional file 1: Table S1. Summary of situations where $\hat{\tau}$τ^ is not calculable or takes very small values (<0.01) among the 64,929 meta-analyses. Table S2. Summary of situations where I2 is not calculable, equals 0%, or takes very small values (≤1%) among the 64,929 meta-analyses. Table S3. Comparisons between I2 of the RD, RR, and OR within the 64,929 meta-analyses. Table S4. Q test results (with the significance level at 0.05) among the pairs of RD, RR, and OR within the 64,929 meta-analyses. Table S5. Summary of descriptive statistics of I2 (%) among the 23,966 meta-analyses with I2>0% for all three effect measures based on both the DL and REML methods. Figure S1. Histograms of between-study standard deviations on a logarithmic scale based on the DerSimonian–Laird method for the RD, RR, and OR. The histograms are restricted to the range from −8 to 2 for log$\hat{\tau}$τ^. Figure S2. Histogram of I2 based on the DerSimonian–Laird method for the RD, RR, and OR, restricted to I2>1% for better visualizations. Figure S3. Boxplots of I2 for the RD, RR, and OR categorized by the number of studies (panels a and b), average study size (panels c and d), and total number of events (panels e and f), restricted to I2>1%. The left panels a, c, and e are based on the DerSimonian–Laird method, and the right panels b, d, and f are based on the restricted maximum likelihood (REML) method. Figure S4. Histogram of CVB on a logarithmic scale based on the DerSimonian–Laird method for the RD, RR, [file 13643_2022_1895_MOESM1_ESM.pdf]

Supplemental File for
“Empirical comparisons of heterogeneity magnitudes of the risk difference, relative risk, and odds ratio”

Table S1. Summary of situations where $\hat{\tau}$ is not calculable or takes very small values (<0.01) among the 64,929 meta-analyses.

**Method**	**Effect measure**	**No. of MAs with REML failing to converge**	**No. of MAs with $\hat{\tau}$<0.01**	**No. of MAs with $\hat{\tau}$≥0.01**
DL	RD	NA	34,284 (52.80%)	30,645 (47.20%)
	RR	NA	36,833 (56.73%)	28,096 (43.27%)
	OR	NA	36,521 (56.25%)	28,408 (43.75%)
REML	RD	35 (0.05%)	35,345 (54.44%)	29,549 (45.51%)
	RR	144 (0.22%)	37,378 (57.57%)	27,407 (42.21%)
	OR	143 (0.22%)	36,879 (56.80%)	27,907 (42.98%)

Note: NA, not applicable; DL, DerSimonian–Laird; MA, meta-analysis; REML, restricted maximum likelihood.

Table S2. Summary of situations where I^2 is not calculable, equals 0%, or takes very small values (≤1%) among the 64,929 meta-analyses.

Method	Effect measure	No. of MAs with REML failing to converge	No. of MAs with I^2=0%	No. of MAs with 0%<I^2≤1%	No. of MAs with I^2>1%
DL	RD	NA	31,222 (48.09%)	219 (0.34%)	31,441 (48.42%)
	RR	NA	36,786 (56.66%)	234 (0.36%)	37,020 (57.02%)
	OR	NA	36,506 (56.22%)	261 (0.40%)	36,767 (56.63%)
REML	RD	35 (0.05%)	28,283 (43.56%)	3,709 (5.71%)	31,992 (49.27%)
	RR	144 (0.22%)	32,718 (50.39%)	4,889 (7.53%)	37,607 (57.92%)
	OR	143 (0.22%)	32,752 (50.44%)	4,368 (6.73%)	37,120 (57.17%)

Note: NA, not applicable; DL, DerSimonian–Laird; MA, meta-analysis; REML, restricted maximum likelihood.

Table S3. Comparisons between I^2 of the RD, RR, and OR within the 64,929 meta-analyses.

Method	Comparison (X vs. Y)	No. of MAs with REML failing to converge in the comparison	No. of MAs with $I_X^2 - I_Y^2$						
			≤−25%	−25% to −10%	−10% to −1%	−1% to 1%	1% to 10%	10% to 25%	≥25%
DL	RD vs. RR	NA	0	0	7,923 (12.20%)	30,131 (46.41%)	9,116 (14.04%)	8,516 (13.12%)	9,243 (14.24%)
	RD vs. OR	NA	0	0	6,538 (10.07%)	31,197 (48.05%)	10,534 (16.22%)	8,562 (13.19%)	8,098 (12.47%)
	RR vs. OR	NA	0	0	15,552 (23.95%)	41,259 (63.54%)	4,610 (7.10%)	1,874 (2.89%)	1,634 (2.52%)
REML	RD vs. RR	176 (0.27%)	0	0	10,041 (15.46%)	29,487 (45.41%)	8,036 (12.38%)	7,093 (10.92%)	10,096 (15.55%)
	RD vs. OR	174 (0.27%)	0	0	8,587 (13.23%)	30,491 (46.96%)	9,373 (14.44%)	7,401 (11.40%)	8,903 (13.71%)
	RR vs. OR	201 (0.31%)	0	0	14,142 (21.78%)	41,455 (63.85%)	5,285 (8.14%)	2,111 (3.25%)	1,735 (2.67%)

Note: NA, not applicable; DL, DerSimonian–Laird; MA, meta-analysis; REML, restricted maximum likelihood.

Table S4. Q test results (with the significance level at 0.05) among the pairs of RD, RR, and OR within the 64,929 meta-analyses.

Pair (X vs. Y)	Q test result based on the effect measure X	Q test result based on the effect measure Y	
		No. of meta-analyses with non-significant Q test result	No. of meta-analyses with significant Q test result
RD vs. RR	Non-significant	50,050 (77.08%)	926 (1.43%)
	Significant	6,615 (10.19%)	7,338 (11.30%)
RD vs. OR	Non-significant	50,391 (77.61%)	585 (0.90%)
	Significant	6,090 (9.38%)	7,863 (12.11%)
RR vs. OR	Non-significant	55,351 (85.25%)	1,314 (2.02%)
	Significant	1,130 (1.74%)	7,134 (10.99%)

Table S5. Summary of descriptive statistics of I^2 (%) among the 23,966 meta-analyses with I^2>0% for all three effect measures based on both the DL and REML methods.

Method	Effect measure	Mean	1st quartile	Median	3rd quartile
DL	RD	58.3	41.5	61.0	77.4
	RR	48.2	29.0	49.1	67.8
	OR	49.7	31.3	50.6	68.4
REML	RD	57.4	38.8	61.9	79.4
	RR	46.7	25.4	48.4	69.3
	OR	48.4	28.9	50.3	69.6

Note: DL, DerSimonian–Laird; REML, restricted maximum likelihood.

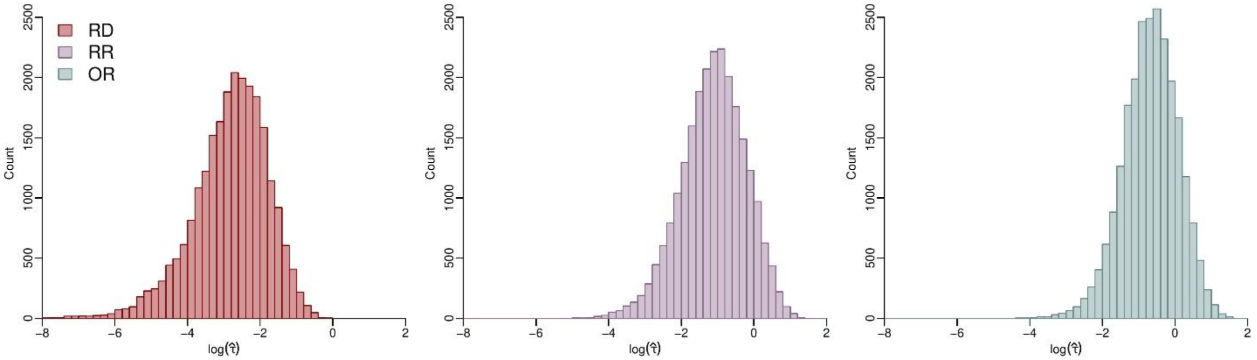

Figure S1. Histograms of between-study standard deviations on a logarithmic scale based on the DerSimonian–Laird method for the RD, RR, and OR. The histograms are restricted to the range from -8 to 2 for $\log\hat{\tau}$.

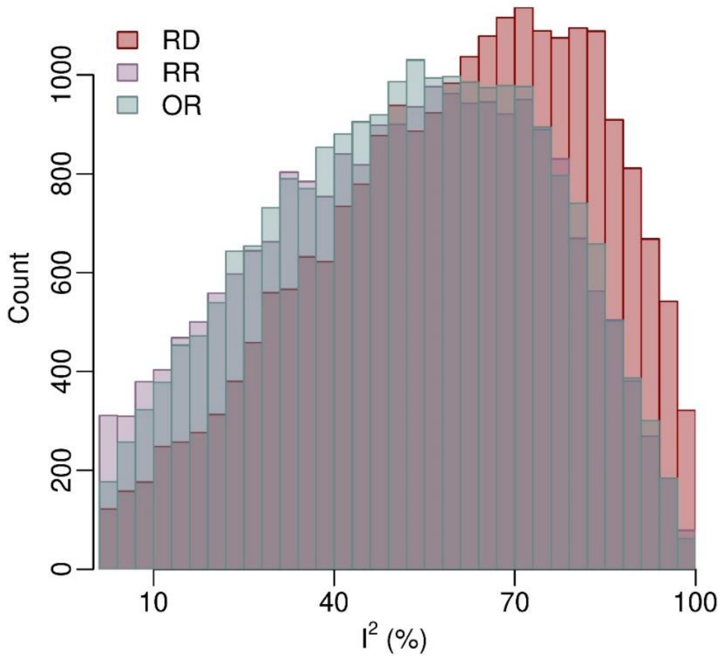

Figure S2. Histogram of I^2 based on the DerSimonian–Laird method for the RD, RR, and OR, restricted to I^2>1% for better visualizations.

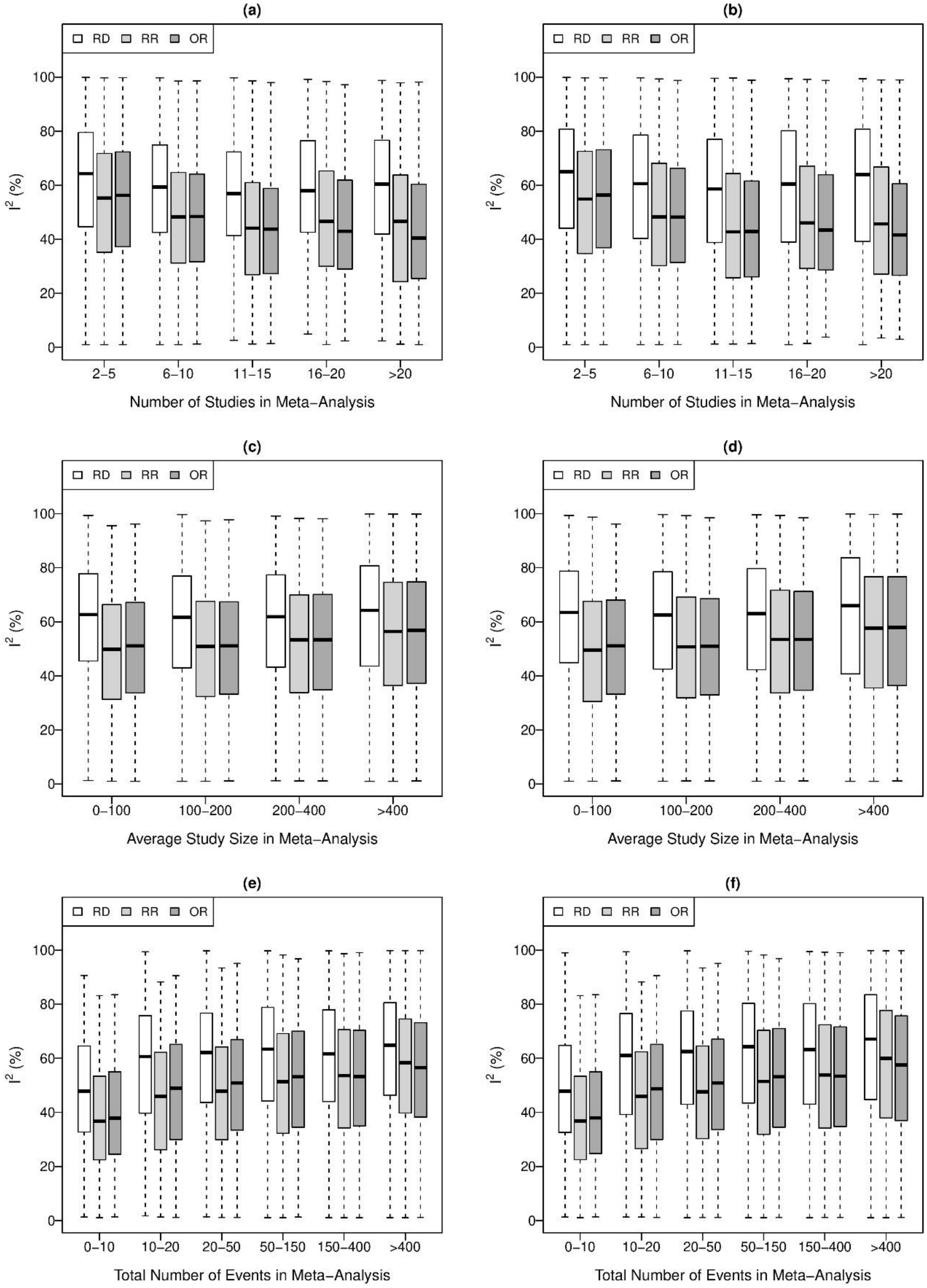

Figure S3. Boxplots of I^2 for the RD, RR, and OR categorized by the number of studies (panels a and b), average study size (panels c and d), and total number of events (panels e and f), restricted to I^2>1%. The left panels a, c, and e are based on the DerSimonian–Laird method, and the right panels b, d, and f are based on the restricted maximum likelihood (REML) method.

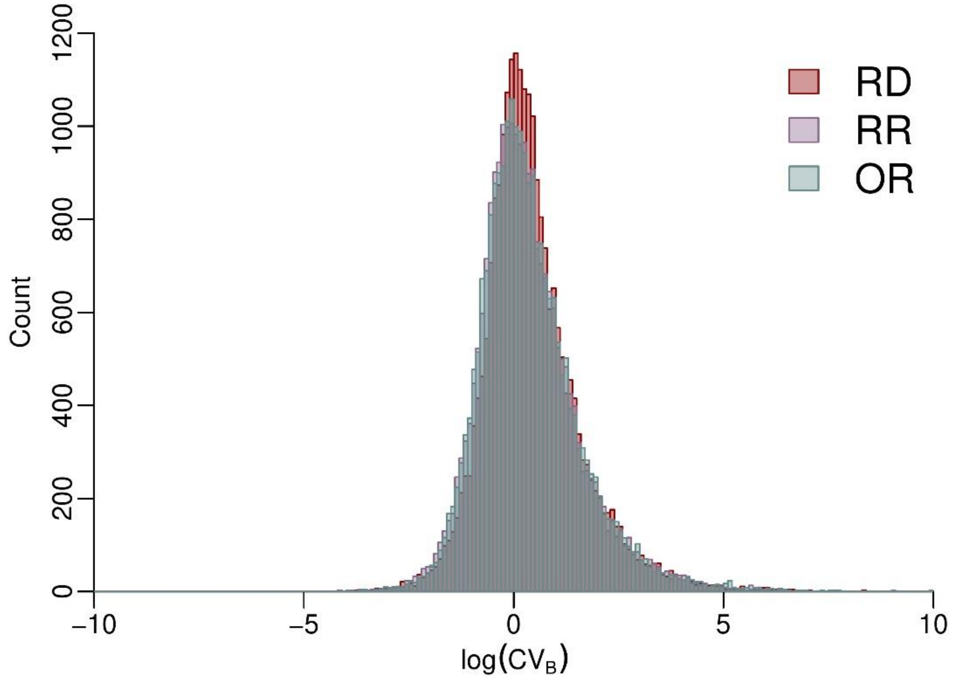

Figure S4. Histogram of CV_B on a logarithmic scale based on the DerSimonian–Laird method for the RD, RR, and OR.

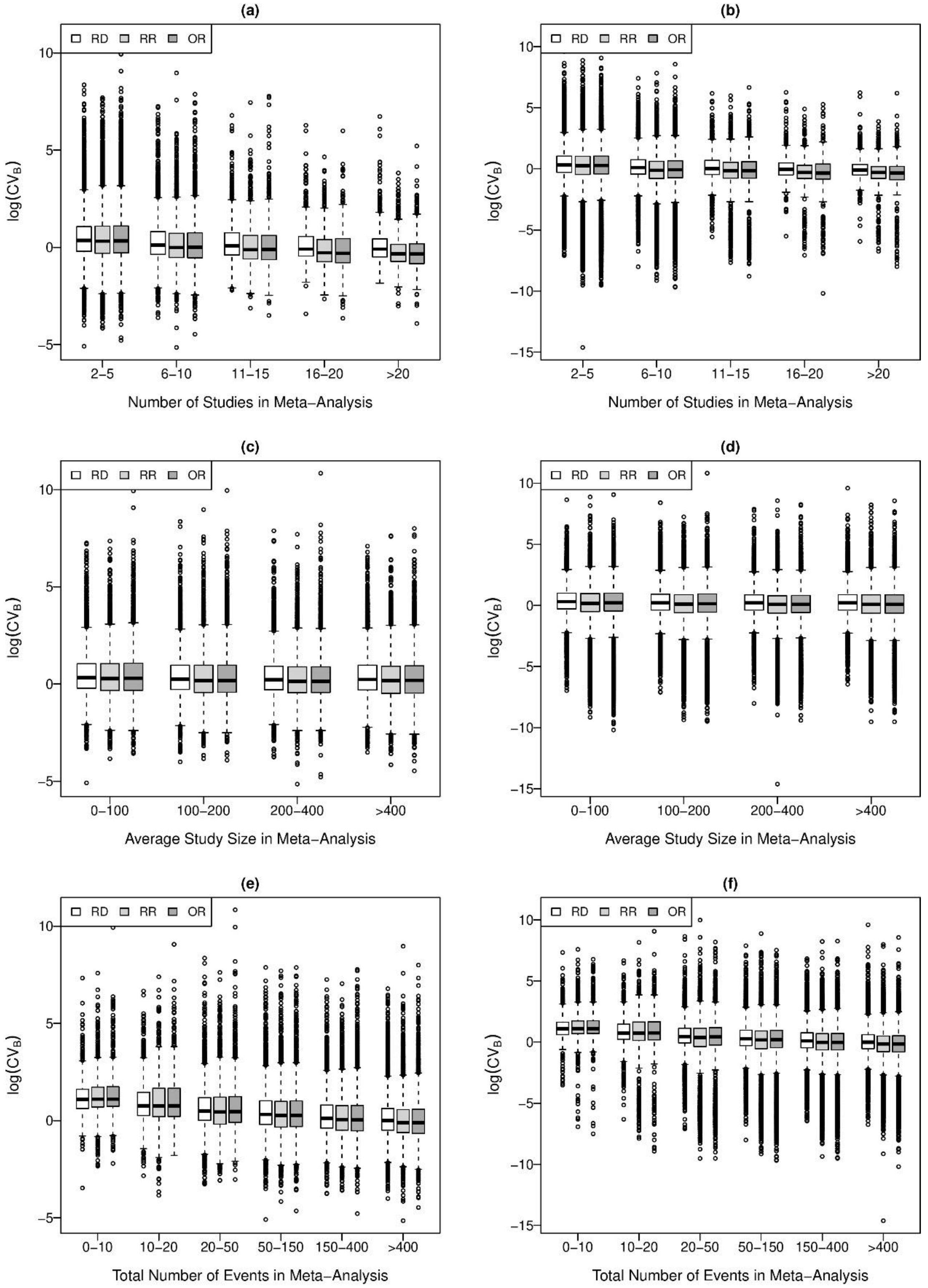

Figure S5. Boxplots of CV_B on a logarithmic scale for the RD, RR, and OR categorized by the number of studies (panels a and b), average study size (panels c and d), and total number of events (panels e and f). The left panels a, c, and e are based on the DerSimonian–Laird method, and the right panels b, d, and f are based on the restricted maximum likelihood (REML) method.

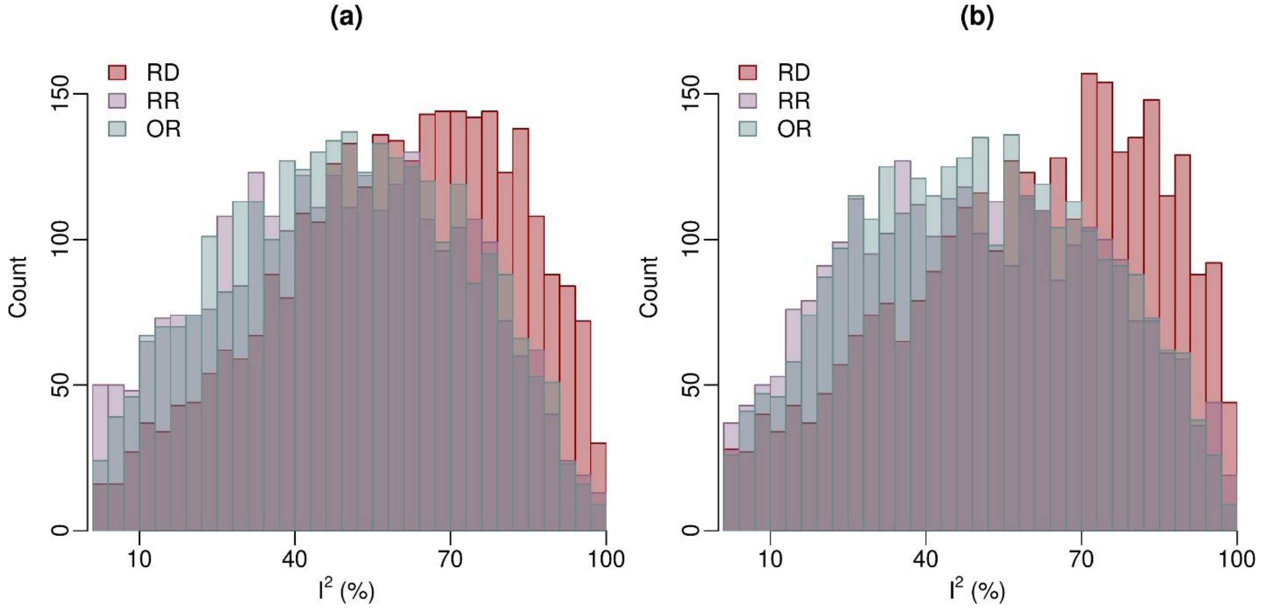

Figure S6. Histograms of I^2 for the RD, RR, and OR, restricted to I^2>1% for better visualizations, among the meta-analyses with the largest number of studies from each Cochrane review. Panel a is based on the DerSimonian–Laird method, and panel b is based on the restricted maximum likelihood (REML) method.

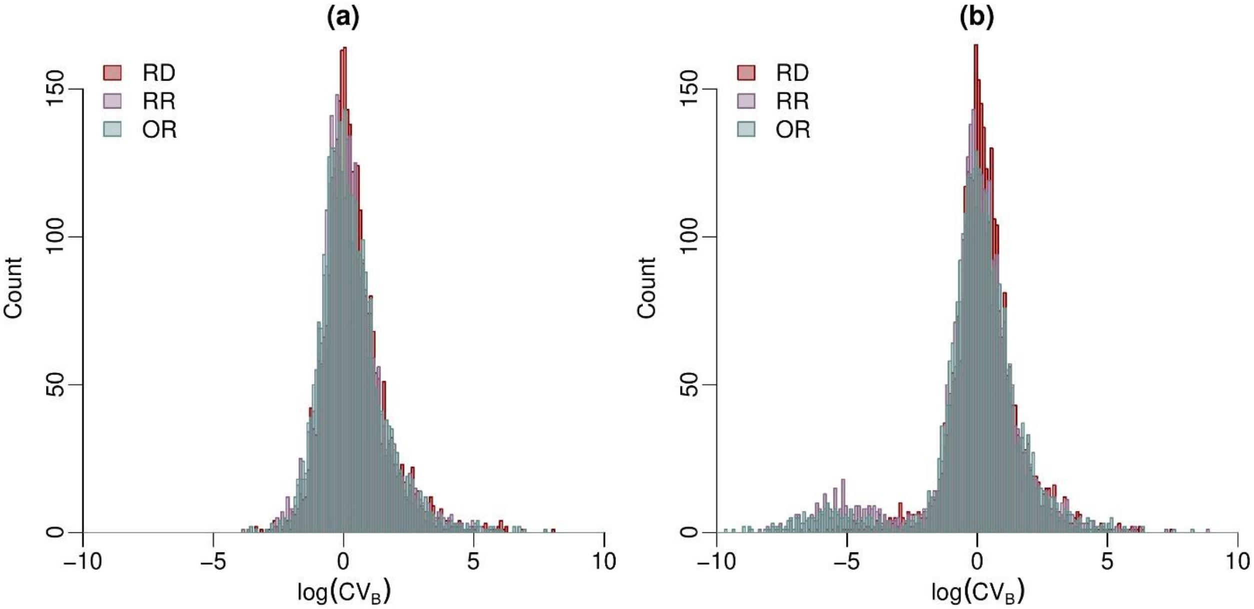

Figure S7. Histograms of $\mathrm{CV_B}$ on a logarithmic scale for the RD, RR, and OR among the meta-analyses with the largest number of studies from each Cochrane review. Panel a is based on the DerSimonian–Laird method, and panel b is based on the restricted maximum likelihood (REML) method.
